# Appendiceal cancer leading to intussusception detected incidentally during follow-up for Peutz–Jeghers syndrome

**DOI:** 10.1007/s12328-020-01200-w

**Published:** 2020-10-09

**Authors:** Kazuhiro Kurihara, Takanori Suganuma

**Affiliations:** Department of Gastroenterology, Iida Municipal Hospital, Iida, Nagano 395-8502 Japan

**Keywords:** Case report, Peutz–jeghers syndrome, Ileocecal carcinoma, Peutz–jeghers-type polyps

## Abstract

Peutz–Jeghers syndrome is an autosomal dominant disorder characterized by hamartomatous polyposis, pigmentation, and malignant tumors. We report a case of ileocecal carcinoma that was incidentally detected during follow-up for Peutz–Jeghers syndrome. A 39-year-old man with solitary Peutz–Jeghers syndrome had undergone three abdominal surgeries. He had been followed up via upper and lower gastrointestinal endoscopy and small intestinal endoscopy. In the endoscopic examination of the lower gastrointestinal tract, a 35 mm large, bumpy, elevated lesion was observed in the cecum. This lesion was not observed 9 months earlier during lower endoscopy. Biopsy of the specimen confirmed tubulovillous adenoma and carcinoma. This lesion was judged to be an indication for operation, and we performed ileocecectomy + D3 lymph node dissection. From the excised specimen, poorly differentiated carcinoma and adenoma components in contact with Peutz–Jeghers-type polyps in the appendix were recognized. A review of the computed tomography image obtained 2 years ago confirmed appendiceal swelling. We suspect that the ileocecal carcinoma in the appendix may have rapidly developed within the 9 months, and was incidentally detected on lower endoscopic examination during follow-up. For the prevention of appendicular tumorigenesis, prophylactic appendectomy may be considered in certain cases during follow-up for Peutz–Jeghers syndrome.

## Introduction

Peutz–Jeghers syndrome (PJS) is characterized by hamartomatous polyposis, pigmentation, autosomal dominant inheritance, and neoplastic transformation derived from Peutz–Jeghers polyposis [1]. We report a case of ileocecal carcinoma detected incidentally during follow-up for PJS.

## Case report

### Chief complaints

A 39-year-old man with PJS reported to our emergency room with watery diarrhea, vomiting, and abdominal distension for several days.

### History of presenting illness

The patient reported that the symptoms of watery diarrhea, vomiting, and abdominal distension had appeared in the beginning of December 2017. When the patient visited a primary care doctor, the leukocyte count and C-reactive protein level were high. Cefmetazole (1 g/day) was administered for 5 days. However, the symptoms worsened, and the patient reported to our emergency room.

### History of past illness

The patient had undergone laparotomy for small intestinal and gastric polyps at the age of 9 and 18 years, respectively. At the age of 33 years, he had undergone forward resection for sigmoid colon cancer: type 1, pap + tub 1, f stage I (pT2pN0cM0) (TNM classification, 8th edition) and had been followed up with upper and lower gastrointestinal endoscopy once a year since then. He had also undergone small intestinal polypectomy at the age of 34 years and endoscopic mucosal resection for colonic polyps at the age of 35 years, and for numerous polyps from the ascending colon to the rectum at the age of 37 years; at both instances, the polyps were Peutz–Jeghers-type hamartomas.

**Fig. 1:**
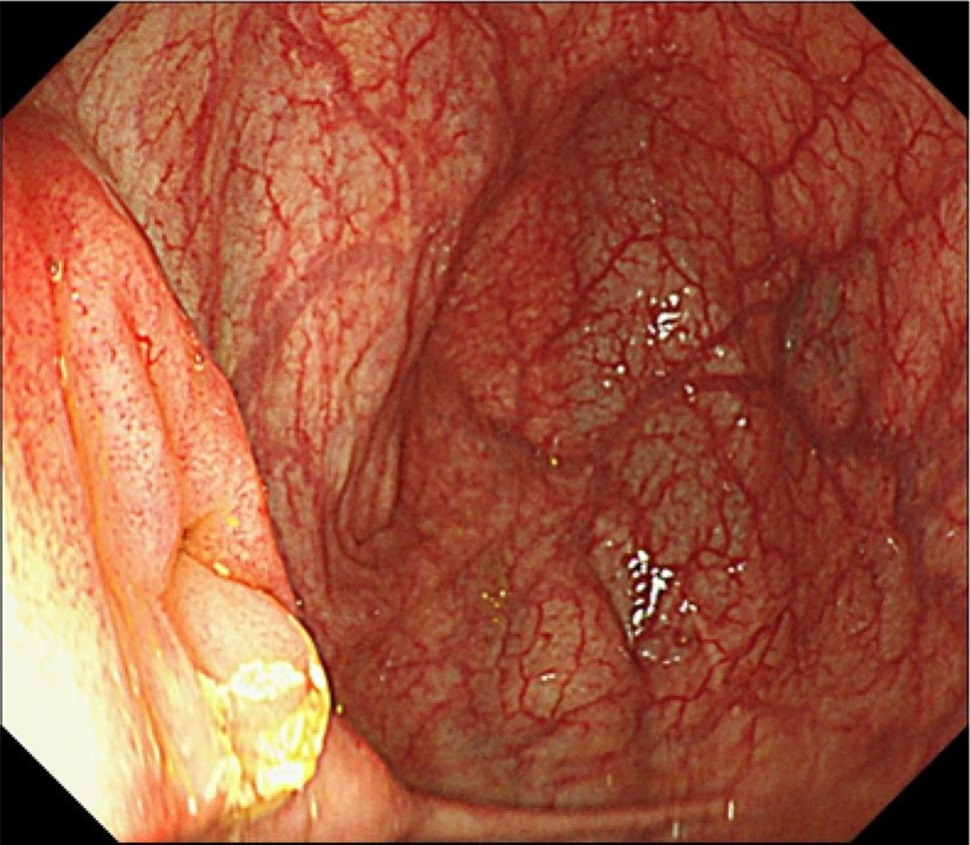
Lower gastrointestinal endoscopy performed in February 2017

In February 2017, lower gastrointestinal endoscopy had been performed, confirming that there was no tumor in the ileocecal area (Fig. 1).

### Physical examination

The patient’s height was 153.6 cm and weight was 52.10 kg. The physical examination further revealed a body temperature of 36.5 °C, pulse of 100 beats/min, blood pressure of 125/77 mmHg, and saturated oxygen of 97% at room air. The eyelid conjunctiva did not indicate anemia, and there was no yellowing of the bulbar conjunctiva. The patient was scarred because of the multiple surgeries. The epicardial region was distorted; however, no tenderness and rebound pain were observed. Tenderness at the McBurney point was evident along with a negative Murphy sign and diminished intestinal movement noise. There was brownish black pigmentation up to 5 mm in size on his face, lip, palm, and fingers.

### Imaging examinations

**Fig. 2:**
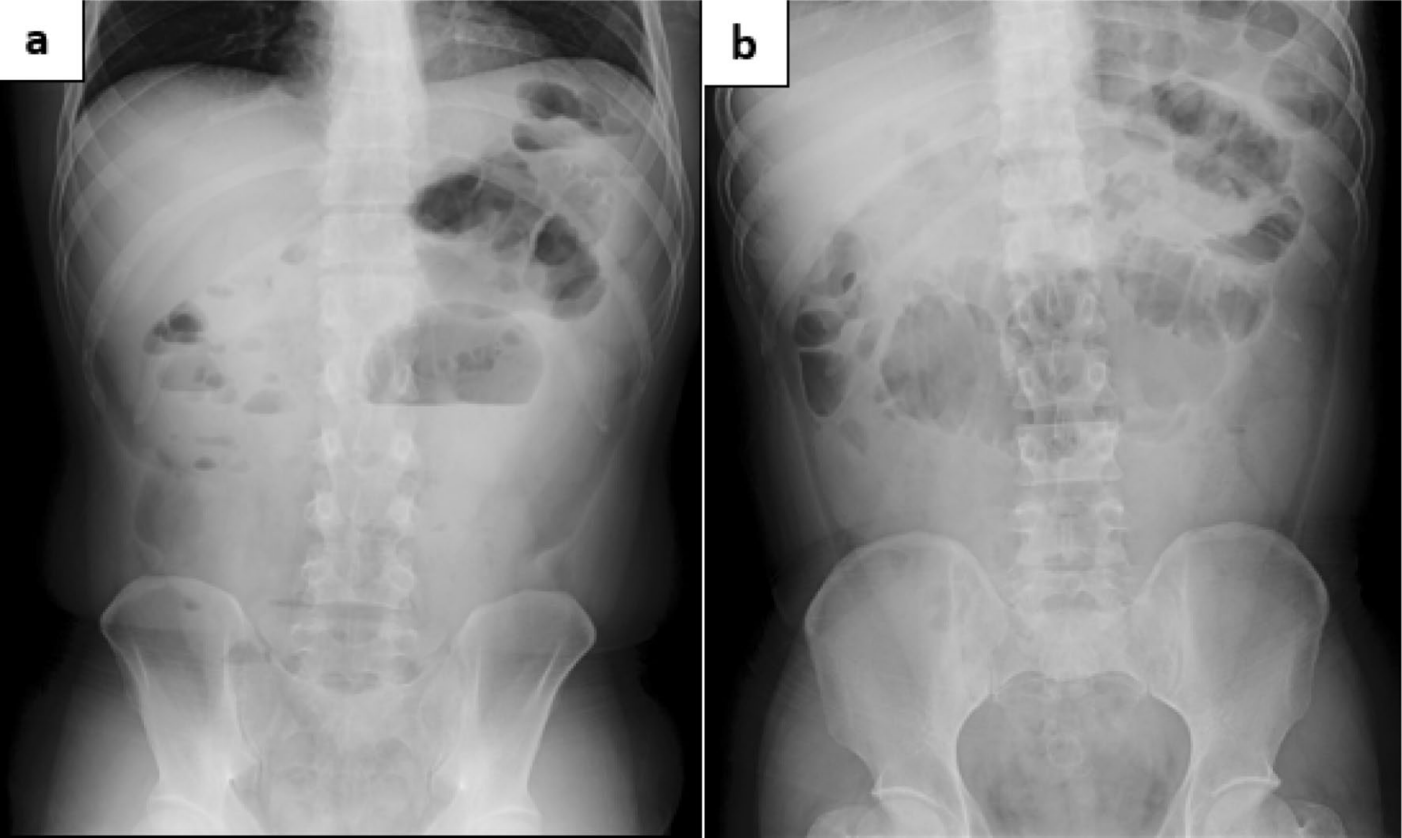
Abdominal radiograph obtained in December 2017. **a** Standing position. **b** Decubitus position

**Fig. 3:**
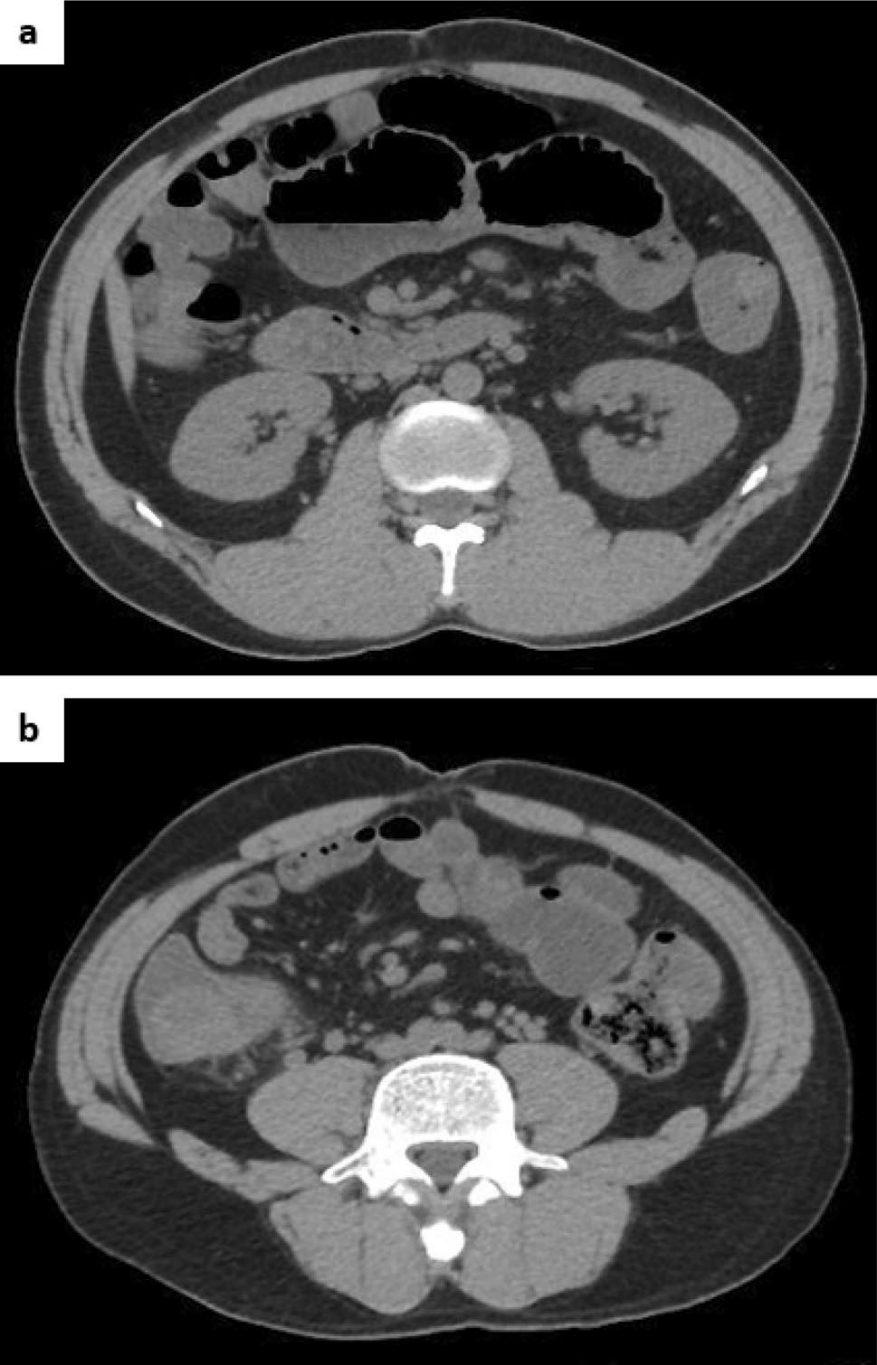
Abdominal plain computed tomography performed in December 2017. **a** Discontinuous dilation in the small and large intestine. **b** Wall thickening of the ascending colon from the cecum near the ileocecal region

**Fig. 4:**
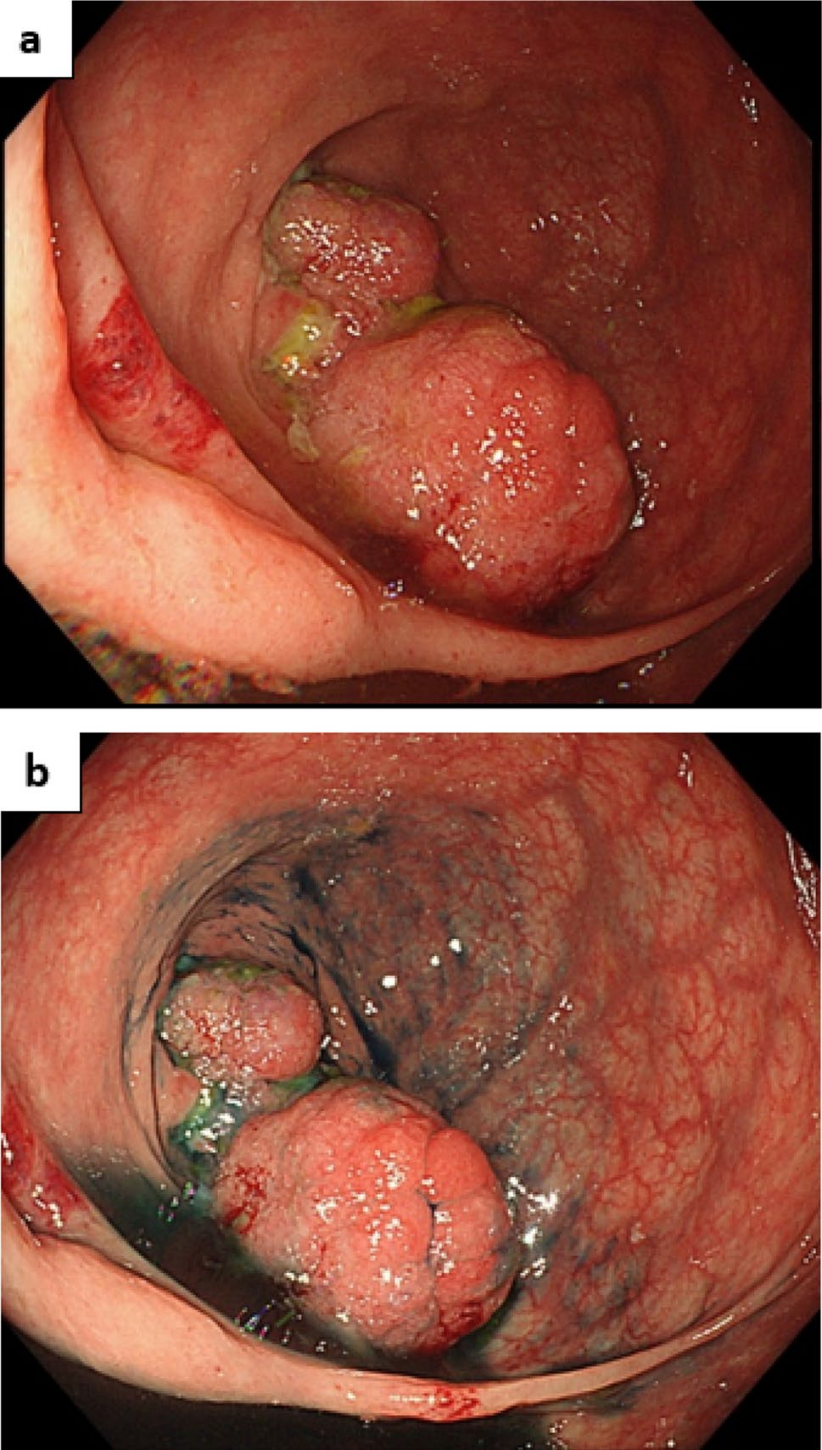
Lower gastrointestinal endoscopy performed in December 2017. **a** Standard image. **b** Image with indigo carmine spray. **c** Narrow-band imaging (NBI) of the appendiceal lesion. **d** NBI of the anal side of the lesion

**Fig. 5:**
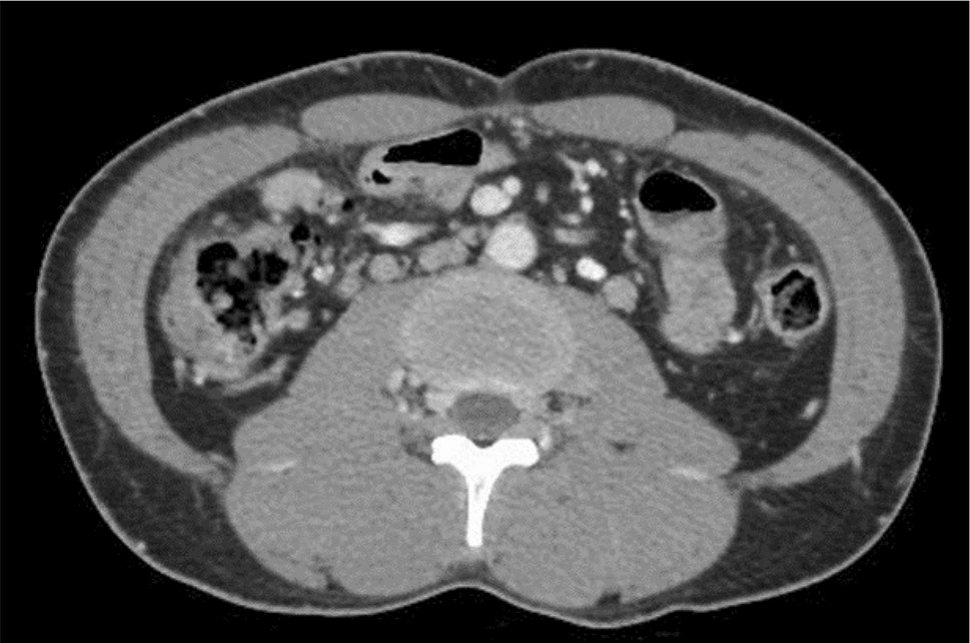
Abdominal contrast computed tomography performed in 2015

**Fig. 6:**
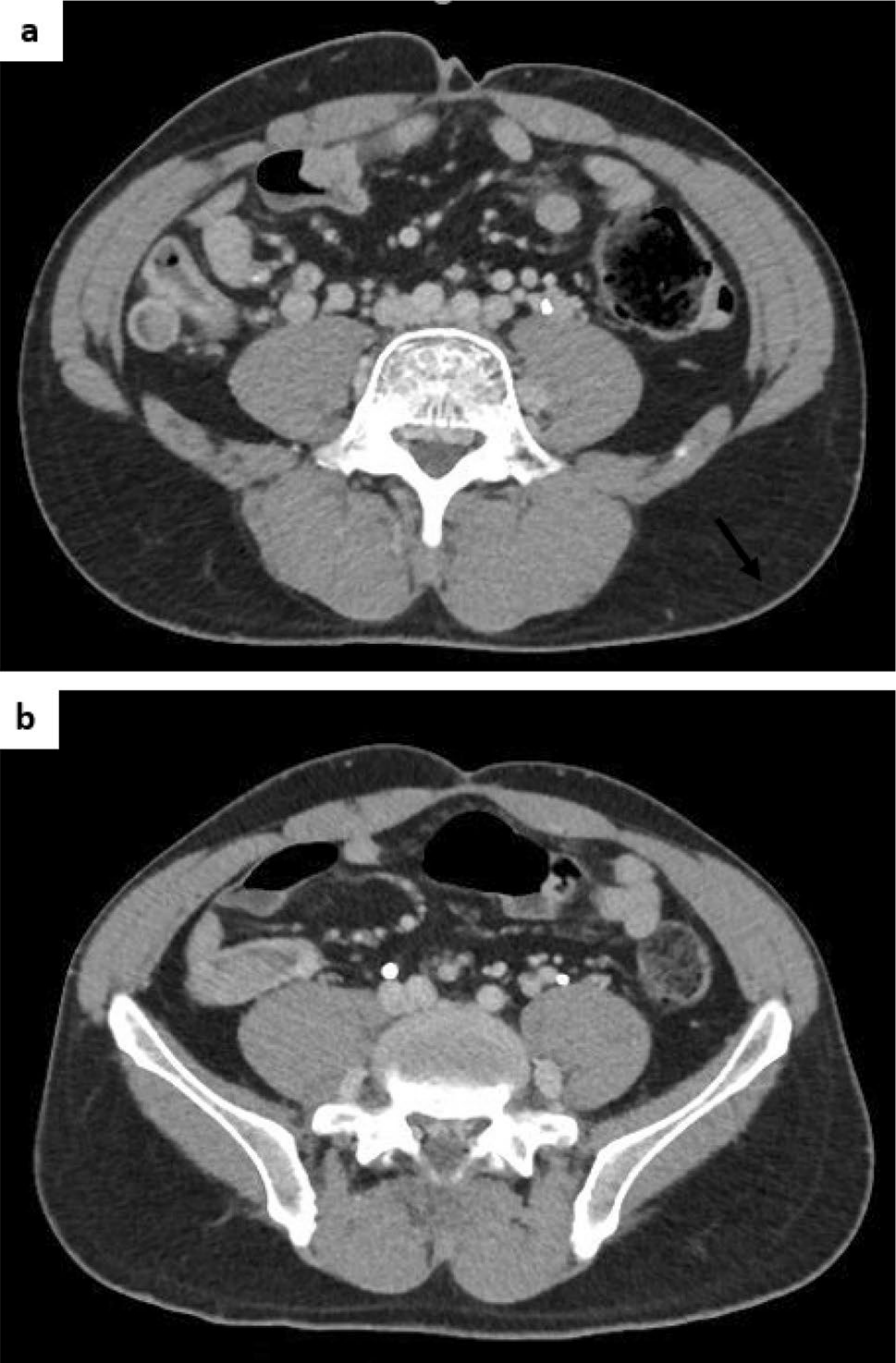
Abdominal contrast computed tomography performed in February 2017. **a**, **b** An enlarged appendix is shown

The niveau sign and gas in the small intestine were noted on an abdominal plain radiograph (Fig. 2a, b). Moreover, thickening of the wall of the cecum and discontinuous dilated regions of the small intestine and colon were seen on abdominal computed tomography (CT) (Fig. 3a, b). Based on these findings, he was diagnosed with paralytic ileus and admitted to our hospital in December 2017. On the third day of hospitalization, we performed a lower gastrointestinal endoscopic examination (Fig. 4) and found a protruding lesion with a 35 mm large, uneven nodule located in the cecum that was not detected in February 2017. The mouth side of the lesion had an expanding appearance, and the appendicular lesion was in contact with the appendicular opening. The surface of the nodule was slightly uneven with a villous change between the elevations (Fig. 4a–c). No obvious irregular blood vessel pattern was observed on narrow-band imaging (Fig. 4c, d). The biopsy result of the ileocecal lesion from the portion between the elevations revealed a high-grade tubulovillous adenoma, while that from the anal side revealed carcinoma of tub 1 and tub 2. We retrospectively examined the CT images from 2015 and noted that the ileocecal portion (Fig. 5) was normal; however, in February 2017 (Fig. 6a, b), CT suggested an enlarged appendix in the portion suspected to be the distal ileum.

## Final diagnosis

The final pathological diagnosis of the main lesion was cecal carcinoma type 1, tub 1 > por 1 > tub 2, f stage IIIc (pT3pN2bM0) (TNM classification, 8th edition).

## Treatment

From the day of hospitalization in December 2017, treatment with antibiotics was started for infectious enterocolitis, and the patient’s diarrhea improved on the second day of hospitalization.

## Outcome and follow-up

**Fig. 7:**
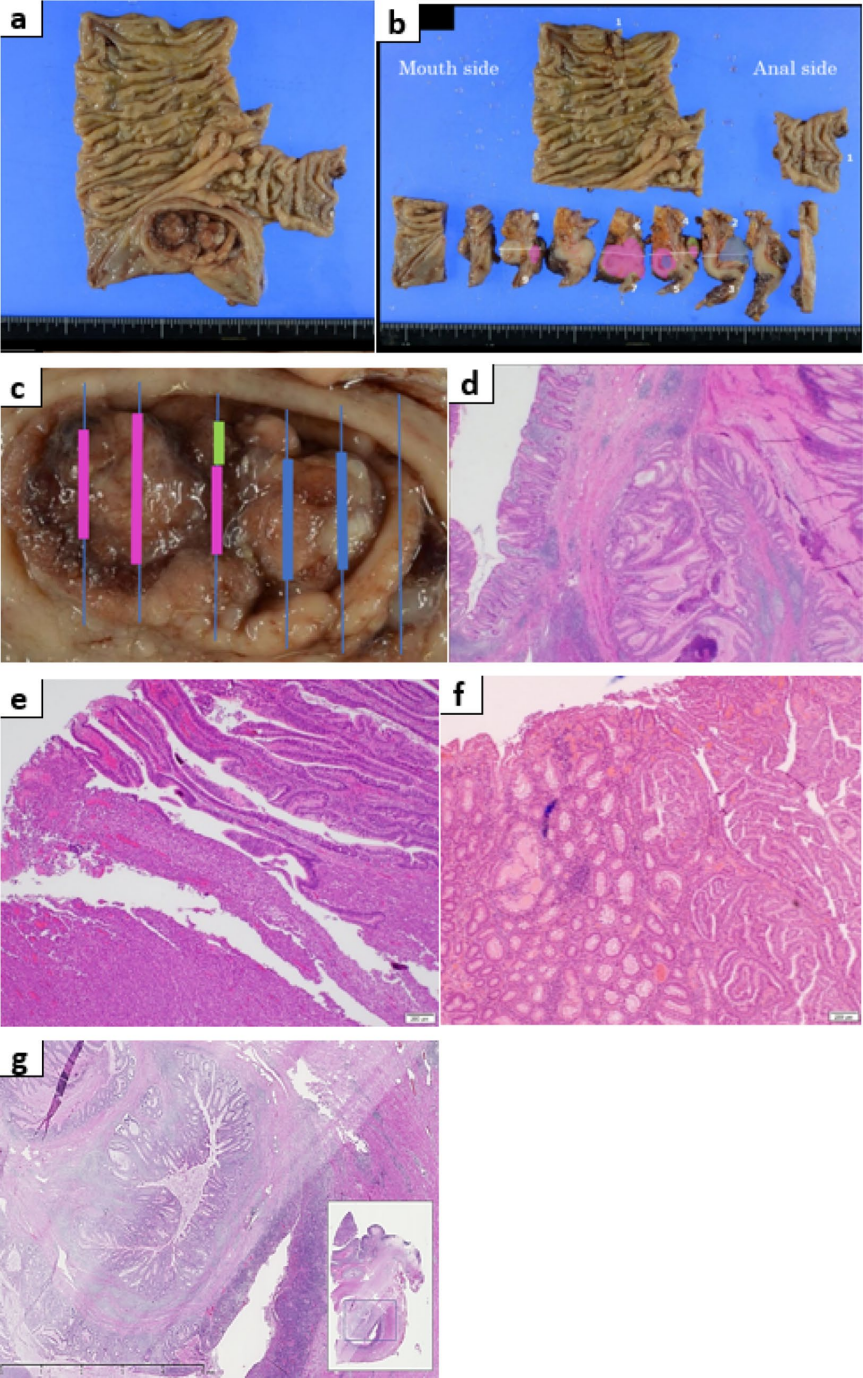
Pathological examination of the resected specimen. **a** Fixed sample. **b**, **c** Peutz–Jeghers-type polyp (blue), adenoma(green), and carcinoma (pink). **d** Peutz–Jeghers-type polyp. **e** Carcinoma (tub 1 + tub 2). **f** Adenoma (left side) + carcinoma (right side). **g** The orifice and stack-like folding of the appendix

In March 2018, ileocecal resection and D3 dissection were performed. The pathological findings of the resected specimen (Fig. 7) and the appendicular lesion nodule (Fig. 4a, b) showed dendritic increase in the muscularis mucosa and hyperplasia of ducts, indicating a Peutz–Jeghers-type polyp (Fig. 7d). The appendicular lesion and that on the anal side were well-differentiated and poorly differentiated adenocarcinomas (Fig. 7e), respectively. Although there was no apparent tissue continuity between the Peutz–Jeghers-type polyps and the adenocarcinomas, some tubulovillous adenoma components were found in contact with the carcinoma. In addition, intussusception to the appendix opening and appendix was observed in the deep layer of the same plane, and Peutz–Jeghers-type polyp-like change was observed in the appendix mucosa (Fig. 7g).

After the third course of capecitabine plus oxaliplatin therapy, no clear sign of recurrence was noted on CT. Recurrence was evaluated based on capsule endoscopy, upper and lower gastrointestinal endoscopic examination, and contrast-enhanced CT.

## Discussion

PJS is an autosomal dominant disease characterized by hamartomatous polyposis occurring in all gastrointestinal tract regions, other than the esophagus, and manifests as pigmented spots on the lips, oral mucosa, and limbs [1]. The causative gene for PJS has been identified as the tumor suppressor gene *STK11/LKB1* [2–4]. The frequencies of occurrence of Peutz–Jeghers hamartomas are 48.0–56.7% in the stomach, 13.8% in the duodenum, 56.9–73.9% in the small intestine, and 48.8–65.8% in the large intestine, and carcinogenesis has reportedly been seen in approximately 3% of cases [5]. Patients with PJS have a high potential to develop malignant tumors of the gastrointestinal tract, breast, pancreas, genital organs, and lungs.

There are various theories regarding the carcinogenic pathway underlying Peutz–Jeghers-type polyps including adenomas occurring in Peutz–Jeghers-type polyps, Peutz–Jeghers-type polyps themselves evolving to be malignant, adenomas existing independently from Peutz–Jeghers-type polyps, and tumors arising in normal mucous membranes of polyps other than Peutz–Jeghers-type polyps [6, 7]. In the present case, adenoma and carcinoma developed concomitantly with the main lesion, and we speculate that a gene mutation had occurred based on the adenoma-carcinoma sequence theory [5].

In the present case, tumorous lesions were not found in the ileocecal region during the lower gastrointestinal endoscopy examination in February 2017, but carcinoma was found in the endoscopy examination in December of the same year. In the specimen resected in March of the next year, carcinoma invading the subserosal layer was observed. If the carcinoma had developed from the mucosa of the ileocecal area and followed the adenoma-carcinoma sequence theory, it seemed unlikely that it could have grown in the short period of approximately 1 year. Moreover, in February 2017, the CT examination showed enlargement of the appendix, suggesting the presence of a tumor in the appendix. Thus, it was speculated that appendix intussusception was present in December 2017; however, since the tumor was hidden in the appendix, it could not be found by lower endoscopy.

Appendix intussusception is a relatively rare disease, and clinical symptoms of abdominal pain and bloody stools are found in approximately half of the cases [8]. Anatomical factors of the appendix and abnormal peristaltic movements are considered to cause its onset [9], and there have been previous reports of PJS manifesting in the appendix [6]. Appendicular intussusception is diagnosed by abdominal ultrasonography, contrast-enhanced enema examination, lower digestive tract endoscopy, and abdominal CT examination; however, preoperative diagnosis is difficult, and many patients are diagnosed by a postoperative pathological examination.

Appendicular lesions of PJS have been reported in nine cases, including our case, until February 2019 based on a PubMed search using the following keyword combinations: “appendix,” “Peutz,” and “Jeghers” [6–8, 10–14]. Among them, there were four cases each of malignant tumor and appendix intussusception (Table 1). In addition, in six of the nine cases, the lesions were detected during surgery or diagnosed based on examination of the resected specimen after surgery. It has been also reported that the preoperative diagnosis of appendiceal cancer is difficult without an imaging modality, such as CT and abdominal ultrasonography [15]. However, in open surgery for conditions such as intussusception in patients with PJS, prophylactic appendectomy can prevent appendicular intussusception and tumorigenesis.

**Table 1:** Cause and frequency of appendiceal lesion in the literature

Author	Year	Age	Complaint	Mallignancy	Appendix intussusception	Chance for discovery
Chang [6]	2014	50	Right lower abdominal pain	−	+	During surgery
Miyahara [7]	1995	40	Anemia	+	+	Contrast enema
Moirangthem [8]	2001	15	Lower gastrointestinal bleeding and rectal prolapse	−	−	During surgery
Yoshikawa [10]	1998	70	Asymptomatic	+	+	During surgery
Skrovina [11]	2007	44	Right abdominal pain	−	−	During surgery
Iida [12]	2008	78	Right lower abdominal pain	−	−	CT
Nozoe [13]	2013	65	Unknown	−	−	Resected specimen
Hofmann [14]	2014	21	Nausea and abdominal pain	+	−	During surgery
Self-experience	2018	39	Vomiting and diarrhea	+	+	CT, colonoscopy

*CT* computed tomography

Recently, Beggs et al. [16] and van Lier et al. [17] conducted surveillance for patients with malignant tumors associated with PJS and proposed algorithms (Table 2); however, no consensus has been reached regarding the PJS surveillance methods and enforcement intervals [18]. Moreover, the proposed surveillance algorithms do not incorporate an imaging modality for organs other than the gastrointestinal tract, such as the appendix. On the basis of the findings in this case, we believe that imaging techniques, such as CT, abdominal ultrasonography, and positron emission tomography, are essential to evaluate the appendix.

**Table 2:** Surveillance of Peutz–Jeghers syndrome

Article	Surveillance algorithm
Begg [16]	Upper and lower digestive tract endoscopy at 8 years
	Polyps detected: every 3 years until 50 years
	Polyps not detected: re-examination at 18 years, after that every 3 years until 50 years
	Lower digestive tract endoscopy: every 1–2 years after 50 years
	Video capsule endoscopy: every 3 years after 8 years
	Testicular ultrasonography: every year until 12 years
	Cervical cytology: every 3 years until 25 years
	Breast MRI: every year after 25 until 50 years
	Mammography: every year after 50 years
Van Lier [17]	Consultation and Hb level test: every year after 10 years
	Upper digestive tract endoscopy: every 2–5 years after 20 years
	Lower digestive tract endoscopy: every 2–5 years after 25–30 years
	Video capsule endoscopy: every 2–3 years after 10 years
	MRCP, EUS: every 2–5 years after 30 years
	Cervical cytology: every year until 25–30 years
	Transvaginal ultrasonography: every year after 25–30 years
	CA-125 level test: every year after 25–30 years
	Breast MRI: every year after 25 years
	Mammography: every year after 30 years

*MRCP* magnetic resonance cholangiopancreatography, *EUS* endoscopic ultrasonography, *CA-125* cancer Antigen 125, *Hb* hemoglobin

In this case, the small intestine was observed using a capsule endoscope, and then polypectomy was performed. Although double-balloon endoscopy is also a useful option for exploration of the small intestine, Ohmiya et al. [19] reported that 90% of patients with a history of less than one laparotomy completed 90% of all small bowel examinations, and for those with a history of laparotomy, they reported a reduction to 27%. Therefore, it is necessary to refer to the history of laparotomy as a means for examining the small intestine, and in this case, we considered that the capsule endoscope was an effective means.

On the basis of our findings, we think that there is a possibility to prevent appendicular tumorigenesis by performing prophylactic appendectomy during follow-up for PJS.
